# Atypical involvement of Alzheimer’s tau proteins in diseases beyond tauopathies

**DOI:** 10.1093/lifemedi/lnag016

**Published:** 2026-05-11

**Authors:** Jie Meng, Zi-Jie Deng, Jing Zhang, Wenxin Yu, Xiaolei Wu, Peng Lei

**Affiliations:** Department of Geriatrics and State Key Laboratory of Biotherapy, National Clinical Research Center for Geriatrics, West China Hospital, Sichuan University, Chengdu 610000, China; Department of Neurology and State Key Laboratory of Biotherapy, National Clinical Research Center for Geriatrics, West China Hospital, Sichuan University, Chengdu 610000, China; Department of Neurology and State Key Laboratory of Biotherapy, National Clinical Research Center for Geriatrics, West China Hospital, Sichuan University, Chengdu 610000, China; Fujiang Laboratory, Southwest University of Science and Technology, Mianyang 621000, China; Department of Geriatrics and State Key Laboratory of Biotherapy, National Clinical Research Center for Geriatrics, West China Hospital, Sichuan University, Chengdu 610000, China; Department of Neurology and State Key Laboratory of Biotherapy, National Clinical Research Center for Geriatrics, West China Hospital, Sichuan University, Chengdu 610000, China

**Keywords:** Tau, multiple sclerosis, diabetes, stroke, breast cancer

## Abstract

Tau is a microtubule-associated protein traditionally involved in a collective group of disorders termed “tauopathy”, including Alzheimer’s disease. Tau protein self-aggregates and forms neurofibrillary tangles in neurons, which are considered a pathological hallmark of tauopathies. While the roles of neuronal tau in tauopathies have been extensively investigated, recent studies have shed light on its roles in other diseases without tau pathology and in other cells. In this review, we aim to discuss the “atypical” pathological involvement of tau in diseases other than tauopathies, including brain diseases (e.g., amyotrophic lateral sclerosis, multiple sclerosis, and spinal cord injury), vascular diseases (stroke and hypertension), diabetes, and cancers. We have discussed the expression and functions of tau in cell types other than neurons, and have summarized the evidence supporting a role of tau in these diseases. These cross-disease studies collectively suggest that tau protein is more broadly implicated in mechanisms such as axonal instability, dysregulated cell signaling, inflammatory activation, and cell death, independent of its aggregation, contributing to our knowledge of the functions of tau and the myriad ways in which it may be involved in pathological processes.

## Introduction

Tau is an essential member of the microtubule-associated protein family and is encoded by a single gene containing 16 exons located on chromosome 17q21 [1, 2]. The gene encoding tau (*MAPT*) is alternatively spliced by RNA, giving rise to six different isoforms in the adult human brain. The protein was initially isolated from the tubulin complex *in vitro* and thought to promote microtubule formation [3], and it may also regulate microtubule-associated axonal transport [4]. However, the survival of mice is not affected by tau loss [5, 6], nor is the rate of axonal transport affected by the alteration of tau expression in the brain [7, 8]. The physiological function of tau *in vivo*, therefore, is still debated.

**Figure 1.**
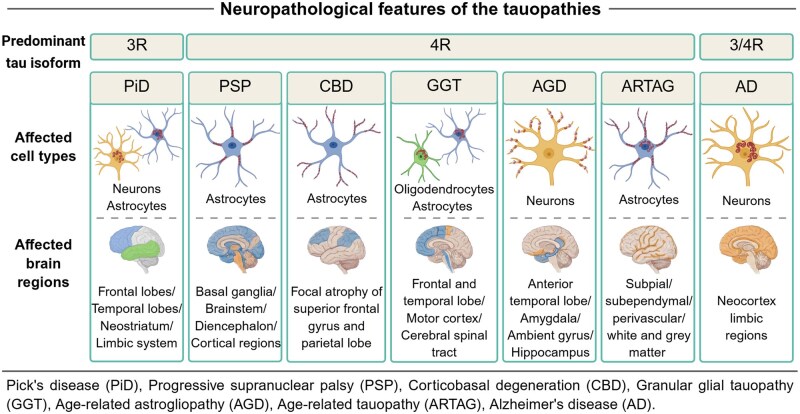
Overview of tauopathies. The tauopathies are categorized by their predominant tau isoform composition (3R, 4R, or mixed 3R + 4R). Below the isoform, the characteristic cellular inclusions are shown in neuronal and glial cell bodies, processes, or astrocytic fibers. The specific brain regions and structures affected in each disorder are also highlighted. The red dots represent the specific locations of fibrils within the cells. PiD, PSP, CBD, GGT, AGD, ARTAG, AD.

Tau was recognized as the main component of neurofibrillary tangles (NFTs) in Alzheimer’s disease (AD) [9–11], and later several other diseases with similar pathologies were identified to be associated with *MAPT* mutations, termed tauopathy [12, 13]. Those diseases are defined by the common pathological hallmark of tau accumulation in the brain. For example, Pick’s disease (PiD) is characterized by a predominance of 3R-tau protein, while a series of diseases, including progressive supranuclear palsy (PSP), corticobasal degeneration (CBD), age-related astrogliopathy (AGD), granular glial tauopathy (GGT), and age-related tauopathy (ARTAG) primarily exhibit 4R-tau protein pathology (Fig. 1). In these diseases, abnormal modifications of tau lead to the formation of insoluble aggregates, causing neuronal dysfunction and death, and subsequently leading to a range of neurological symptoms.

Among these post-translational modifications, phosphorylation plays a pivotal role physiologically and pathologically in tau [12]. In normal physiological conditions and concentrations, tau is an unaggregated, highly soluble, unfolded protein. However, tau hyperphosphorylation has been identified to facilitate tau aggregation, which transforms it into a folded, insoluble protein [12]. It should be cautioned that NFTs are not the result of tau phosphorylation alone, and other factors may contribute to tau aggregation, such as tau acetylation [1], ubiquitination dysfunction [14, 15], and environmental factors such as biometals [16, 17]. Furthermore, specific phosphorylation of tau is involved in the pathogenesis of AD [18, 19] and may be used as a biomarker for AD in cerebrospinal fluid (CSF) and plasma [20–25].

**Figure 2.**
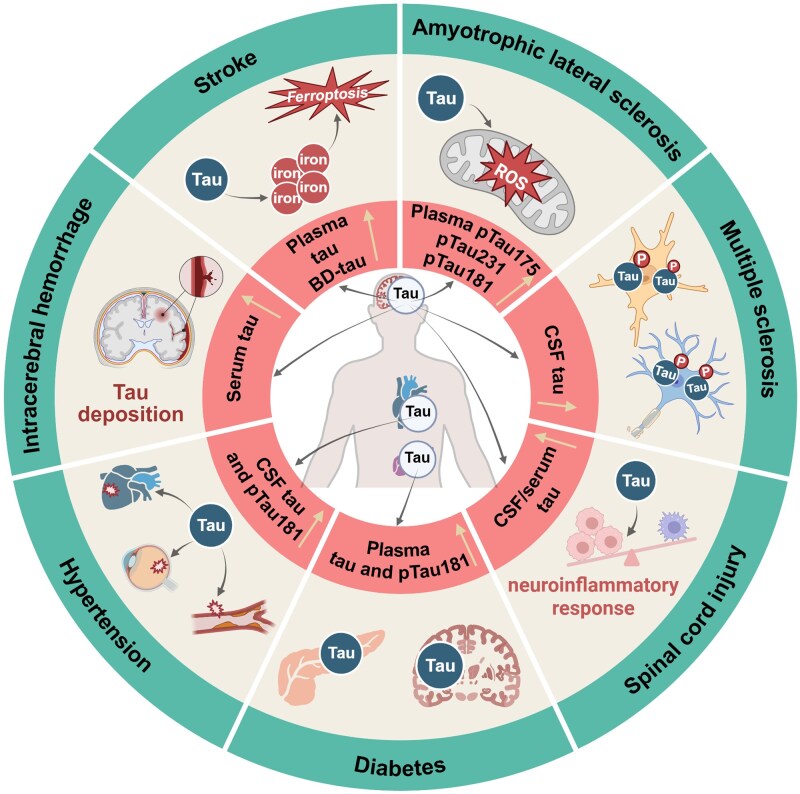
An overview of tau protein across neurological and systemic diseases. Tau and phosphorylated tau (p-Tau) levels in plasma, serum, or CSF correlate with disease severity and outcomes in stroke, intracerebral hemorrhage (ICH), hypertension, diabetes, amyotrophic lateral sclerosis/frontotemporal dementia (ALS–FTD), multiple sclerosis (MS), and spinal cord injury (SCI). Tau is involved in cellular activities of iron metabolism, excitotoxicity, insulin regulation, mitochondrial dysfunction, neuroinflammation, and remyelination. Tau may be a potential biomarker and therapeutic target in these disorders.

On the other hand, tau protein is somewhat less recognized as a regulator of disease processes. In fact, in several neurological disorders where tangles were not commonly seen, or even in diseases that mainly affect other organs than the brain, the tau protein was found to be crucial in the pathogenesis [26–29]. In this review, we elaborate on the broad pathological involvement of tau protein beyond the scope of traditional tauopathies, encompassing various domains such as brain disorders, vascular diseases, metabolic diseases, and oncological diseases (Fig. 2). As a key regulator of cellular homeostasis, tau was found to be involved in maintaining iron metabolism balance in the brain [30], regulating central and peripheral insulin and glucose metabolism [31], and participating in cardiovascular functions [29], extending the commonly recognized roles in neurons. Furthermore, tau protein demonstrates significant potential as a biomarker for diagnosis, subtyping, and prognosis assessment in subtypes of cancers [32–35]. Collectively, the evidence repositioned tau protein as a central molecular node within multisystem and multi-organ pathophysiological networks, which should be considered as a critical target with substantial value for cross-disease precision diagnosis and targeted therapy development.

## Tau in brain cells

**Figure 3.**
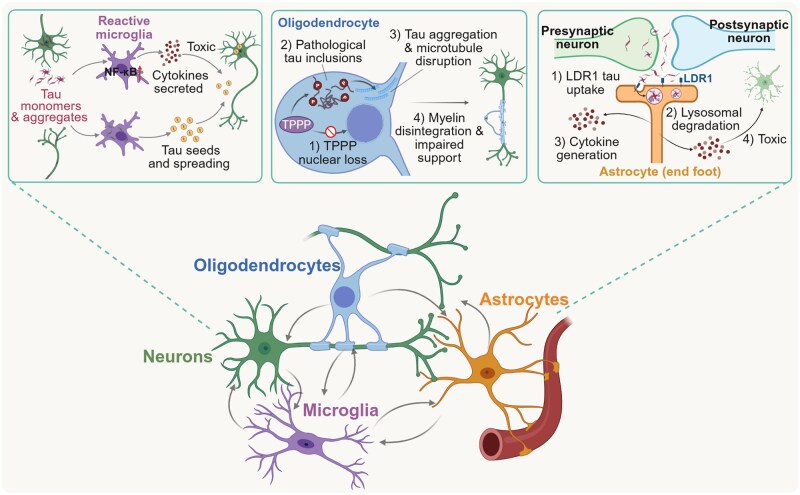
Tau is physiologically and pathologically involved in non-neuronal brain cells. Microglia facilitate tau spread via exosomes. Astrocytes contribute to tau hyperphosphorylation and propagation through inflammatory signaling. Oligodendrocytes exhibit disrupted homeostasis under tau pathology, characterized by loss of nuclear tubulin polymerization-promoting protein (TPPP) expression, cytoplasmic swelling, and myelin disintegration. Tau, therefore, mediates a pathogenic network of cell–cell interactions among glia and neurons, driving neuroinflammation, synaptic loss, and disease progression.

It is noted that tau is traditionally considered a marker for neurons [1, 36]. Still, recent evidence indicates that other types of cells in the brain are also directly or indirectly involved in tau functions and dysfunctions (Fig. 3).

Microglia, the resident immune cells, can be pathologically activated and execute neuroinflammation or phagocytic activities [37]. It was generally believed that tau protein in microglia mainly originated from the uptake of neuronal tau and did not possess significant tau expression capabilities. Secreted tau^2N4R^ does not cause neuronal death directly, but instead promotes microglial uptake of stressed neurons, resulting in gradual neuronal loss [38]. After taking up neurons with tau aggregates, microglia become hypophagocytic, release tau aggregates capable of seeding, and display a senescence-like phenotype, thereby acting as vectors of tau aggregate spreading [39]. In a mouse model of tau propagation, microglia promote tau spread via exosome secretion, and eliminating microglia or inhibiting exosome synthesis suppresses the process, demonstrating that microglia act as *in vivo* vectors of tauopathy [40]. Consistently, another study showed that microglia from adult rTg4510 mouse brains or AD brains released tau seeds, indicating their incomplete neutralization of seeding activity [41]. Further investigation revealed that exosomes released by TREM2-deficient microglia had a stronger tau seeding capacity, exacerbating the spread of tau pathology both *in vivo* and *in vitro* [42]. Microglial nuclear factor kappa B (NF-κB), a key transcription factor regulating neuroinflammation, has been reported to drive tau spreading and toxicity in a mouse model of tauopathy [43].

Notably, a recent study unexpectedly indicated that microglia possess the ability to endogenously express both the *MAPT* gene and tau protein. By analysis of RNA-seq data from microglia isolated from the human brain, robust *MAPT* mRNA expression in all samples was identified. Subsequently, using induced pluripotent stem cells (iPSCs) from patients with the *MAPT* IVS10 + 16 mutation and their syngeneic controls, microglia-like cells (iMGLs) were differentiated, and they showed detectable full-length tau protein, supporting that microglia can be an endogenous source of tau protein [44]. This is puzzling, but single-cell RNA sequencing can detect low levels of RNAs in microglia, which are usually not detectable at corresponding protein levels [1]. Tau protein may have high turnover rates or low translation efficiency, resulting in this consistency. As for the expression of tau protein in iMGs models derived from human iPSCs, there are also issues. For example, iPSC-derived cells usually retain epigenetic characteristics similar to those of a fetus [45–47], and their endogenous tau protein expression may reflect a transient developmental state rather than a stable physiological condition of a mature adult brain. Future research should utilize a microglia-specific *MAPT* conditional knockout model to thoroughly define the relative contributions *in vivo*.

Astrocytes connect with neurons, coordinating synaptic activities [48], and take up extracellular tau protein through receptor-mediated endocytosis, ultimately degrading and clearing it in lysosomes. Although astrocytes express tau protein at low levels under physiological conditions, pathological processes or altered gene expression can lead to its upregulation. For example, in PSP, *MAPT* mRNA levels are increased in astrocytes containing tau protein inclusion bodies, indicating that they are actively expressing tau protein [49]. Early studies found that reactive astrocytes (GFAP-positive astrocytes) contain phosphorylated tau protein (Ser396 and Ser404) [50]. Subsequently, in human AD patients, 3R tau accumulation was observed in astrocytes in the hippocampus, specifically the hilus of the dentate gyrus, which led to mitochondrial dysfunction, neurodegeneration, and spatial memory impairment [51]. Large-scale population cohort studies further demonstrated that astrocyte responsiveness is crucial for triggering Aβ-induced tau phosphorylation, and inhibiting astrocyte responsiveness alleviates tau pathology [52]. In animal models, PS19 mouse astrocytes exhibited the ability to take up pathological tau and degrade it lysosomically, a process regulated by histone deacetylases [53]. Similarly, knocking out the circadian rhythm protein BMAL1 activated astrocytes and enhanced their phagocytosis of tau protein [54]. Notably, astrocytes may have multiple roles in tau pathology. During neuroinflammation, activated astrocytes secrete the chemokine CXCL10, a key driver of intraneuronal tau protein hyperphosphorylation and synaptic damage [55]. Tau-containing astrocytes directly induced tau protein lesions in healthy neurons [56], indicating a role of astrocytes in tau spreading.

Oligodendrocytes possess a complex microtubule network, providing a structural basis for organelle trafficking and intracellular translocation of myelin. Early *in situ* hybridization studies showed that tau mRNA in the adult human cerebral cortex and hippocampus was mainly localized in neurons and not detected in glial cells [57]. However, several subsequent studies have reported low levels of tau mRNA expression in cultured oligodendrocytes [58–61], *in situ* and *in vivo* oligodendrocytes. A study using tauGFP knock-in/knock-out mice reported endogenous tau expression in both oligodendrocytes and neurons [62]. More recently, a study using RNAscope combined with immunohistochemistry systematically mapped and quantified *MAPT* expression across different brain regions and cell types in the adult human brain [63]. This study detected *MAPT* mRNA in neurons, oligodendrocytes, and astrocytes, with the highest density of *MAPT* transcripts in neurons, followed by oligodendrocytes and astrocytes [63]. Notably, tau-immunopositive inclusions in oligodendrocytes can still be observed in brain regions lacking obvious neuronal tau [64, 65], suggesting that oligodendrocytes may utilize endogenous tau protein. In disorders such as PSP, tau pathology in oligodendrocytes, astrocytes, and neurons exhibits differentiated hierarchical patterns [66, 67], also suggesting that glial tau inclusions can develop independently of neuronal tau. The impact of pathological tau aggregation on oligodendrocyte function may involve the disruption of multiple cellular pathways, ultimately impairing their supportive function for neurons. In a cohort of GGT, PSP, multiple system atrophy (MSA), and Lewy body disease (LBD), oligodendroglial tau pathology is most prominently associated with loss of nuclear TPPP/p25α immunoreactivity in GGT, where tau inclusions strongly correlate with such loss as well as with myelin degeneration and microglial reaction. In PSP, oligodendroglial tau pathology is also present but less characterized, and in MSA and LBD, oligodendroglial α-synuclein pathology predominates over tau, highlighting disease-specific relationships between oligodendroglia and tau versus other proteinopathies[68].

Tau may also mediate cell–cell interactions in the brain. For example, the interaction between astrocytes and microglia is part of a complex innate immune response [69], which may be exacerbated by tau aggregation. Cross-sectional studies have shown that both microglia and astrocytes can abnormally phagocytose synapses in the brains of dementia patients, which may be driven by the accumulation of tau oligomers in synapses [70]. Another study found a significant interaction between plasma GFAP (an astrocyte activation marker) levels and plasma p-Tau217 levels in subjects with positive microglial activation [71]. This suggests that microglia, astrocytes, and synaptic tau oligomers interacts synergenerstically in the early development of AD. CellChat analysis revealed that abnormal tau protein induces abnormal slit guidance ligand 2 (SLIT2) signaling from excitatory neurons to oligodendrocytes, and DAP12 (DNAX-activation protein 12, highly and selectively expressed in microglia) deletion blocked tau-induced alterations in microglia, neurons, and oligodendrocytes [72]. These findings suggest that tau protein not only functions within neurons but also actively regulates the crosstalk between microglia, astrocytes, oligodendrocyte, and neurons, forming a network of cellular interactions that drives disease progression.

## Tau in other brain disorders than tauopathies

While tau is the defining pathological hallmark of tauopathies, its involvement in the central nervous system extends significantly. Here, we discuss amyotrophic lateral sclerosis (ALS), MS, and SCI as prime examples of “atypical tau involvement.” These diseases were selected since the formation of classical tau protein deposition was not their primary pathology, and there is emerging evidence that tau protein is involved in these contexts. By examining these non-tauopathy brain disorders, we highlight the broader role of tau as a core mediator of cell death and a potential therapeutic target across a wide spectrum of neurological conditions.

### Amyotrophic lateral sclerosis

ALS is a neurodegenerative disease characterized by the loss of spinal motor neurons. Even normally without tangle presentation [73], all six tau isoforms can be detected in the spinal cord of ALS patients, with elevated p-Tau175 and p-Tau231 detected [74, 75]. Elevated plasma p-Tau181 was found to be associated with ALS clinical outcomes, including weight loss, reduced forced vital capacity, and lower ALS functional rating scale (ALSFRS-R), indicating that p-Tau181 may serve as a diagnostic and prognostic biomarker for ALS [76]. Furthermore, clinical data suggest that more than half of ALS patients exhibit frontotemporal dementia (FTD), accompanied by cognitive and language impairments, collectively referred to as frontotemporal spectrum disorders of ALS (ALS-FTSD), in which tau plays a mediating role in the pathological process [77]. The mechanisms underlying tau-mediated ALS pathology are poorly understood. One study demonstrated that elevation in p-Tau396 increased oxidative stress and induced mitochondrial fragmentation, leading to neuronal dysfunction and exacerbating ALS pathology in mice [78]. On the other hand, it is proposed that tau interacts with RNA-binding proteins, which triggers nuclear dysfunction and results in motor neuron death, while reducing tau pathology effectively alleviates ALS pathology [79].

### Multiple sclerosis

MS is another neurodegenerative disease characterized by neuroinflammation, axonal injury, and subsequent progressive neuronal loss [80]. Although elevated CSF tau levels in MS have been demonstrated in multiple studies [81–85], the role of tau protein as a pathological marker of MS needs further in-depth research. One study investigated the tau phosphorylation in MS patients, reporting widespread abnormal tau hyperphosphorylation of the classic tau phospho-epitopes (AT8, AT180, and AT100) occurring in both glia and neurons with an astrocyte predominance [86]. On the other hand, analysis of CSF tau in MS patients at different disease stages revealed a high level of tau, even in the early stage, without distinction between different MS subtypes [87, 88]. In animal models of MS, tau may be involved in the experimental autoimmune encephalomyelitis (EAE) process via promoting microtubule stabilization, since mice lacking tau exhibited increased synapse loss and activated NF-κB pathway after EAE [89]. More interestingly, in human P301S tau transgenic mice, hyperphosphorylated tau induces early axonal injury and a mild inflammatory environment, which in turn primes oligodendrocyte progenitor cells (OPCs) to differentiate more efficiently when demyelination occurs, leading to increased oligodendrocyte density and myelin basic protein within lesions. However, P301S tau axons are inherently more vulnerable to demyelination-induced degeneration, and therefore, the enhanced OPC differentiation fails to translate into successful remyelination, allowing the ongoing tau pathology to promote toxic tau aggregation and axonal loss [90].

### Spinal cord injury

SCI is a severe central nervous system disorder resulting in various secondary pathological changes following the initial physical impact on the spinal cord, where tau was considered a biomarker for neuronal damage. Tau protein can be detected in the CSF and serum in the early stages of SCI, and the elevated tau positively correlated with injury severity [91]. Fully injured individuals exhibited higher CSF p-tau/tau ratios compared to healthy or incompletely injured individuals [92]. Follow-up studies revealed significantly reduced levels of tau in the CSF among those who exhibited significant improvement in their condition [93].

In rats with SCI, injection of tau siRNA alleviated neuroinflammation and oxidative stress, promoted neurogenesis, and facilitated functional recovery [94]. Immunotherapy using anti-*cis*-p-Tau antibodies after SCI also alleviated subsequent pathological changes. The elimination of pathogenic *cis*-p-Tau via systemic administration of appropriate monoclonal antibodies restored the motor dysfunction, memory impairment, and abnormal risk-taking behavior in SCI mouse models [95]. It was suggested that tau hyperphosphorylation following SCI interfered with neural stem cell migration efficiency, thereby affecting the recovery process [96], which may be mediated by asparagine endopeptidase activation [97].

### Similarities and differences of tau involvement between AD and neurodegenerative diseases without tangle presentation

AD and various neurodegenerative diseases (such as FTD, Parkinson’s disease (PD), and other tauopathies) exhibit complex commonalities and differences, both mechanistically and pathologically. The core differences lie in the morphology, distribution, and the role of tau aggregates in the diseases. For example, AD is characterized by the formation of NFTs composed of highly phosphorylated tau within neurons, which extend along specific brain regions [98, 99]. In other neurodegenerative diseases, tau abnormalities may manifest in several ways different than AD. For example, in frontotemporal degeneration, tau may predominate in the 3R or 4R subtypes, forming non-tangled aggregates [100]. In PD, tau abnormalities may be secondary to other major pathologies (such as α-synuclein) and not a primary driver of the disease [101]. In CBD, tau pathology may be confined to specific brain regions (such as cortical basal ganglia) and may not form typical NFTs [102]. On the other hand, the similarities include the presence of abnormal hyperphosphorylation, conformational changes, and intracellular mislocalization of tau protein. Dysfunctions of tau protein (such as decreased microtubule stability and impaired neuronal transport) are also a shared pathogenic factor in these diseases [103, 104]. Furthermore, tau pathology spreads in these diseases, which drives pathological progression [39, 105]. Therefore, pathological differences in diseases did not alter the downstream toxic pathways, highlighting the role of tau as a core mediator of cell death.

Blood is the most common biological fluid, and has been utilized in tau-related disease research [106]. The plasma levels of amyloid-beta (Aβ)42/Aβ40 and p-Tau, especially p-Tau181, p-Tau217, and p-Tau231, have shown potential for early diagnosis of AD [107]. Plasma tau can differentiate between AD and its control group, but it cannot distinguish AD from other neurodegenerative diseases, such as PD, Lewy body dementia, and FTD [108–110]. Plasma tau shows significant overlap among different disease groups and does not have a linear relationship with the concentration of tau in CSF within each group [111]. The levels of tau in the blood dramatically increase in diseases such as brain injury, stroke, and Creutzfeldt–Jakob disease [112–114]. The specific results of measuring the atomic molar concentration of tau subtypes in plasma by mass spectrometry indicate that only one-fifth of plasma tau comes from the brain, while the rest is of peripheral origin [115]. These findings suggest that the main source of plasma tau is peripheral, rather than the central nervous system. However, further research indicated that the brain-derived tau (BD-tau) in plasma may be a reliable biomarker for AD, as it is closely related to the severity of AD and distinguishable between neurodegenerative diseases [116].

**Figure 4.**
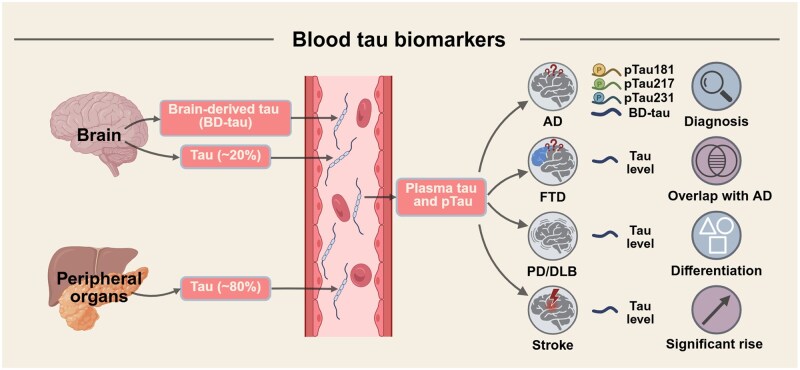
Overview of blood tau biomarker research. Highlighting biomarker profiles in AD, FTD, Parkinson’s disease/dementia with Lewy bodies (PD/DLB), and acute neurological injuries. AD: Plasma phosphorylated tau isoforms (p-Tau181, p-Tau217, p-Tau231) demonstrate high potential for early diagnosis and monitoring, with BD-tau emerging as a specific neurodegeneration marker. FTD: Plasma total tau levels often overlap with AD; currently, no highly specific blood tau biomarker exists for FTD differentiation. PD/DLB: Total tau levels show mild elevation but are generally lower than in AD, limiting the differentiation capacity of conventional plasma tau. Stroke: Characterized by a significant acute-phase rise in plasma total tau that correlates with injury severity.

Currently, phosphorylated tau proteins in the plasma, such as p-Tau181 and p-Tau217, have shown translational value in the early diagnosis and disease monitoring of AD [117]. However, routine blood tau testing still has limited ability to differentiate AD from other neurodegenerative diseases (such as FTD and PD), mainly due to the pathological overlap between different diseases (Fig. 4).

## Roles of tau protein in vascular diseases

### Stroke

Ischemic stroke is a complex neurological syndrome due to limited blood supply in the brain, accounting for 85% of all stroke cases [118]. Oxygen and nutrient deprivation caused by cerebral ischemia and subsequent ischemia-reperfusion damage to brain tissue affect tau protein expression and its post-translational modifications [119], but it is rarely reported that tau deposition would present in the brains of stroke patients. However, levels of different forms of tau protein may serve as biomarkers to predict clinical indications after stroke. A prospective cohort study with 10 years of follow-up demonstrated a nonlinear association between baseline plasma tau levels and stroke risk. Higher tau levels significantly increased the risk of ischemic stroke, with an adjusted hazard ratio of 1.68 (95% CI: 1.18–2.40) [120]. In studies of acute ischemic stroke (AIS), plasma tau levels exhibited a dynamic profile that correlated closely with clinical symptoms, where they surged initially and then stabilized [121]. In patients with successfully recanalized AIS, plasma tau levels between treatment and 24 h showed moderate predictive capacity for clinical outcome (AUC = 0.71, *P *< 0.05) [122]. Further studies have utilized a highly specific assay that selectively measures BD-tau but not tau potentially produced by peripheral tissues, and found that plasma BD-tau levels and cerebral infarct volumes were highly correlated, and the elevated plasma BD-tau in the acute phase was significantly associated with adverse functional outcomes after ischemic stroke, with the highest discriminative performance for 90-day functional outcomes (AUC = 0.76, *P *< 0.001) [123–126]. In addition, p-Tau181 shows a unique association with post-stroke cognitive impairment (PSCI): increased plasma p-Tau181 levels are associated with a reduced risk of PSCI, and the levels are highest in patients without PSCI, followed by patients with late-onset PSCI, and lowest in patients with persistent PSCI, suggesting that it may be used for PSCI course classification [127].

**Figure 5.**
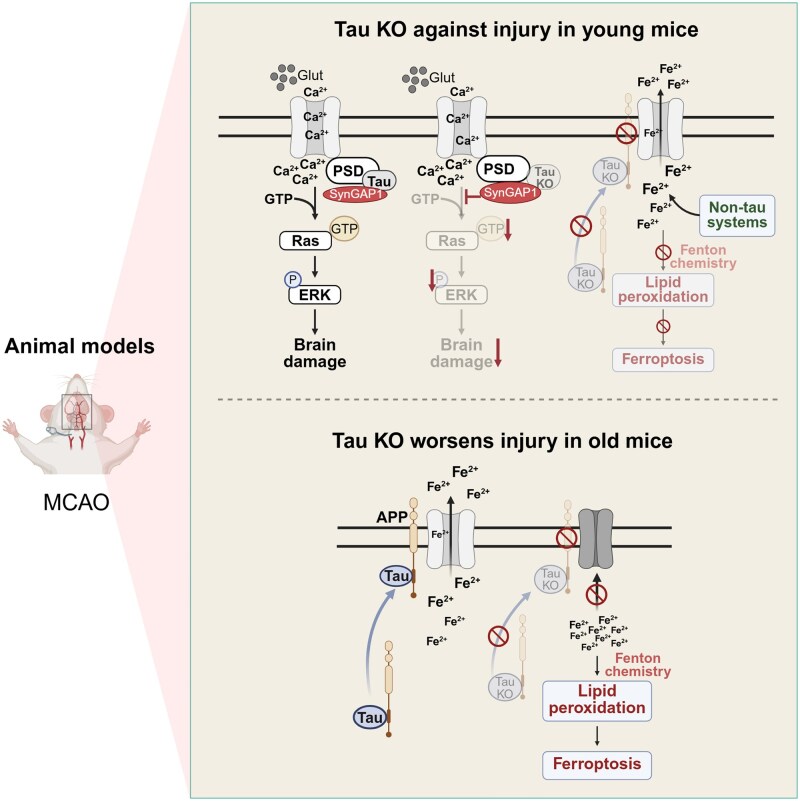
The role of tau protein in stroke. Tau deficiency is protective in young mice but exacerbates injury in old mice. In young mice (3 months), tau knockout confers significant protection against injury. In contrast, aged mice (12 months) lose this protection due to cerebral iron accumulation, which creates a susceptibility to ferroptosis. Restoring iron export or inhibiting ferroptosis rescues the protective phenotype in aged tau-deficient mice. Meanwhile, tau reduction upregulates SynGAP1, suppressing overactive RAS–ERK signaling, thereby mitigating excitotoxic neuronal injury. These pathways highlight that tau is implicated in stroke-related cell death pathways by promoting ferroptosis and excitotoxicity.

Consistently, tau is implicated in the cell death pathways during ischemic/reperfusion in animal studies. Tau knockout mice exhibited a significant protective effect against ischemic stroke in 3-month-old mice. In contrast, the protection was absent in 12-month-old mice, and iron status in the brain was considered responsible [128]. Previous studies indicated that old tau knockout mice accumulate iron in the brain [6], which provides an environment that is susceptible to ferroptosis, a non-apoptotic cell death pathway that involves iron, oxygen, and oxidizable phospholipids [129]. Following-up experiments indicate that the promotion of iron export or inhibition of ferroptosis restores the protective effect of tau knockout in aged mice, confirming an important role for tau-mediated iron ion levels in ischemic stroke [128]. Additional subsequent studies have also shown that thrombin is involved in lipid metabolic processes and mediates reperfusion-related injury through ACSL4-dependent ferroptosis in ischemic stroke, again illustrating the central role of iron levels as well as ferroptosis in ischemic stroke [130]. Spontaneously, tau reduction may also affect excitotoxicity in stroke mice. In an ischemic stroke event, the level of excitotoxicity was upregulated, and knockout of tau reduces the excitotoxic response by increasing the level of SynGAP1, an inhibitory regulator of excitotoxic RAS–ERK signaling, which in turn protects against the injury [131]. These studies not only highlighted the close mechanistic relationship between AD and ischemic stroke through tau, but also pointed out the potential of tau as a therapeutic target to treat ischemic stroke (Fig. 5).

### Intracerebral hemorrhage

Intracerebral hemorrhage (ICH) is another common subtype of stroke, with high rates of disability and mortality. The predominant etiologies of ICH encompass arteriolosclerosis, cerebral amyloid angiopathy, and macrovascular structural pathologies [132]. ICH can lead to an increase in tau protein levels in the brain and blood. Studies have found significant tau accumulation in the ipsilateral Meynert basal ganglia in patients with large infarcts in the middle cerebral artery supply area or putaminal hemorrhage [133]. In patients with ICH, serum tau protein concentration has been proven to be an independent predictor of 3-month mortality and adverse functional outcomes, with predictive power comparable to the National Institutes of Health Stroke Scale score [134]. Another independent study showed that tau protein levels were significantly elevated in the perihematoma region within 60 h after ICH [135]. These pieces of evidence collectively indicate that ICH can not only induce local tau protein pathological deposition, but also that changes in peripheral tau levels are closely related to the severity of the acute phase and long-term neurological outcomes.

### Hypertension

Hypertension, the vascular disease primarily affecting the heart, may also be associated with tau protein. Midlife hypertension increases the risk of dementia in late life [136]. Late development of midlife hypertension, persistent hypertensive state, or late-life hypotension is associated with an increased risk of dementia [137, 138]. An association between hypertension and cognition was proposed to be mediated by tau pathology, independent of amyloid pathology, and this association had a significant age correlation, with middle-aged hypertension being a more risky factor for cognitive impairment and tau pathology compared with old age [139]. In cognitively normal older adults, tau and p-Tau181 in CSF of hypertensive patients increase more rapidly over time [140].

In mice, tau protein is expressed in cardiac tissue [29, 141–143], and tau knockout mice presented age-dependent increased systolic blood pressure and cardiac hypertrophy, indicating the functional role of tau in the cardiovascular system and the negative consequences of its reduction [29]. In triple-transgenic mice of AD, hypertensive modeling increased the levels of p-Tau412 compared to controls and promoted learning memory impairment [144]. In P301L mice, the hypertensive modeling resulted in motor dysfunction [145]. Age-related increase in retinal tau occurs in a rat model of high intraocular pressure glaucoma, and reduction of tau levels by the siRNA ameliorates the damage [146]. These studies suggest that hypertension may mediate the development of tau pathology. In addition, hypertension-mediated tau pathology may be associated with ApoEε4, since tau accumulates more rapidly in ApoEε4 carriers who have hypertension [147, 148]. These studies collectively provided insight into the peripheral function of tau protein, and it should be cautioned to systematically reduce tau as a therapeutic strategy.

## Tau protein in metabolic diseases

Diabetes mellitus is the most common metabolic disease and is mainly categorized into type 1 and type 2 diabetes mellitus (T1DM and T2DM). Patients with T1DM frequently display cognitive dysfunction, and T2DM is associated with an increased risk of developing AD and, because of insulin resistance, leads to NFTs and the formation of amyloid plaques in the AD brain [149–151]. On the other hand, patients with AD also exhibited a higher risk of developing T2DM, and in a community-based cohort study, the prevalence of T2DM increased from 18.1% to 34.6%, and the prevalence of insulin resistance from 23.8% to 46.2%, in patients with AD compared to healthy controls [150]. Hyperphosphorylation of tau protein in neurons in the brains of AD patients led to insulin accumulation and caused insulin resistance, which also supports an association between T2DM and AD [152]. A small cohort study demonstrated significantly elevated plasma total-tau levels in diabetic patients compared with controls [153]. Subsequent cohort studies further showed that in patients with T1DM, higher plasma p-Tau181 levels were associated with greater blood glucose excursions and were significantly positively correlated with glycated hemoglobin (HbA1c) [154]. Elevated p-Tau181 levels were also negatively correlated with psychomotor function and working memory performance [155]. However, no significant association was found between elevated p-Tau181 levels and cognitive decline, mild cognitive impairment, or suspected dementia risk. In an analysis of p-Tau217, higher plasma p-Tau217 was associated with a longer duration of diabetes [154–156]. However, an observational cohort study based on African–American adults did not find a significant association between diabetes and plasma p-Tau217 [157].

In a PET imaging study of a subset of dementia associated with T2DM metabolic abnormalities, only 40% of subjects were PiB (amyloid) positive, whereas 91% were PBB3 (tau) positive, indicating that diabetes-related dementia is more likely to be associated with tau pathology [158]. In animal studies, diabetic status was consistently reported to affect the phosphorylation of tau proteins in the brain. In the streptozotocin (STZ) induced T1DM monkey model, the phosphorylation of tau at ser396/404 was increased in all regions of the brain [159]. Increased phosphorylation of tau in the brain was also detected in the STZ-induced T1DM mouse model, and in the cerebral cortex and hippocampus of the *db*/*db* T2DM mouse model, with an age-dependent increase in tau cleavage [160]. It has been shown that age-increased tau hyperphosphorylation in the brain of T2DM mice correlated with the level of ubiquitination, that p-Tau212 and p-Tau231 in neurons were strongly co-localized with ubiquitin and negatively correlated with P62, a known cargo molecule that transports polyubiquitinated tau to proteasomal and autophagic degradation systems [161].

Considering all the observations, one mechanistic hypothesis to link the diseases together is that tau affects glucose metabolism in the brain. Indeed, in the model of T1DM induced by STZ, reducing tau significantly rescued the cognitive and behavioral deficits compared to the STZ-injected control mice, suggesting that tau protein is the key molecule for STZ-induced cognitive impairment in T1DM [162]. More importantly, tau has been proposed to be a key protein in insulin regulation in the brain, whereas tau knockout mice exhibited insulin resistance in the hippocampus, which may be via the dysfunction of Insulin Receptor Substrate 1. The same mice prevented the anorexigenic effects of cerebral insulin administration, leading to metabolic disturbances [163].

Another possible explanation is that tau acts in the pancreas directly. It was previously shown that aggregated Aβ and hyperphosphorylated tau can be detected in pancreatic Langerhans islets from T2DM patients [164]. In the tau knock-in mouse model, more disturbed glucose metabolism was observed in mice given a high-fat diet, which was associated with an increased number of β-cells in mouse pancreatic islets, and the immunofluorescence analysis showed that tau protein was enriched in the β-cells [165]. Further analysis revealed that overexpression of tau in the β-cell-derived rodent cell line Rin-5F reduces insulin secretion [27], highlighting the possibility that regulation of tau affects insulin metabolism in the pancreas. Consistently, enhanced insulin release and increased insulin sensitivity in tau knockout mice were observed compared to control, and tau reduction in the pancreatic islet β-cell line INS-1 also promoted insulin secretion [31]. Nocodazole-induced microtubule disassembly promotes insulin release [166], and tau knockout decreases the extent of microtubule aggregation in *db*/*db* T2DM model mice, suggesting that tau protein inhibits insulin release from pancreatic islet cells by promoting microtubule assembly [31]. Previous studies have also shown that microtubule stability acts on the regulation of insulin metabolism, and that tau mediates insulin secretion by modulating microtubule dynamics [167]. More importantly, such functions of tau observed in the pancreas may be disturbed in AD, as the correlation between plasma tau and glucose in healthy controls was lost in AD [31]. These results highlight that pancreatic tau may be a therapeutic target for T2DM treatment and acts as a bridge between the pancreas and the brain.

**Figure 6.**
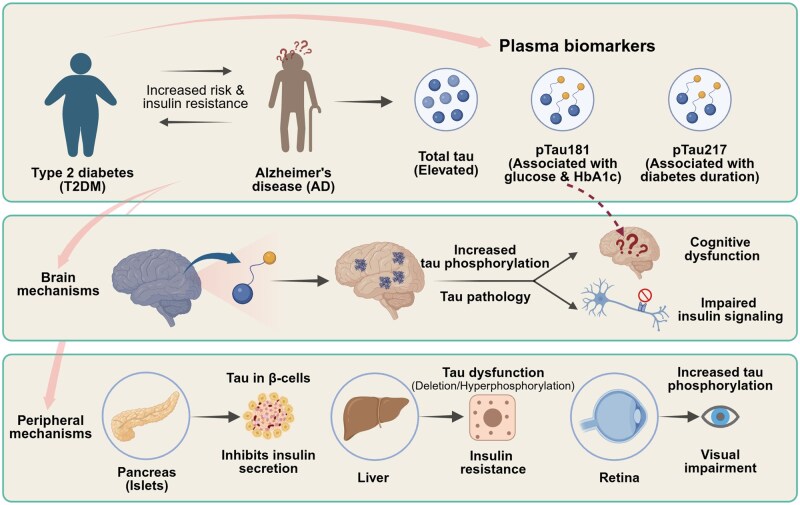
Association between type 2 diabetes and tau pathology. Elevated plasma levels of total tau, p-Tau181, and p-Tau217 are linked to diabetes duration, poor glycemic control, and increased Alzheimer’s disease risk. Tau dysfunction, including hyperphosphorylation and altered expression, impairs insulin signaling and secretion in peripheral tissues such as pancreatic β‑cells and the liver, promoting systemic insulin resistance. In the brain and retina, tau pathology further disrupts neuronal insulin signaling, contributing to cognitive decline and visual disturbances.

In addition to the brain and pancreas, tau was shown in other organs in diabetes. Phosphorylation of tau was increased in the retina of a mouse model of diabetes induced by a high-fat diet, and intravitreal knockdown of tau restored the visual impairment and synaptic damage [168]. In primary hepatocytes from tau knockout mice, P301L mice, and control mice, only primary hepatocytes from control mice did not exhibit insulin resistance, suggesting that both deletion and hyperphosphorylation of tau affect insulin metabolism in the liver [169], both of which can be considered loss-of-function (Fig. 6).

The above studies have demonstrated that tau may be functional in both the central nervous and peripheral systems in diabetes mellitus, and exploring the differences of tau between the central and peripheral systems, as well as elucidating the mechanisms of action, are crucial for understanding diabetes mellitus and its link with AD.

## Tau protein in cancers

Identified and investigated as a neuronal protein, tau was not expected to be associated with cancer. However, emerging evidence indicates that tau may be correlated with the sensitivity of chemotherapy drugs in cancers. For example, paclitaxel is associated with tau expression in the tumorous tissue. Paclitaxel is reported to interfere with spindle microtubule dynamics due to binding to beta-tubulin, causing cell cycle arrest and apoptosis. Tau, meanwhile, as one of the microtubule-associated proteins, may interact with paclitaxel due to binding to beta-tubulin at the same binding site, and consequently, compete with the drug and influence its sensitivity [170–172]. Consistently, several clinical studies identified that the expression levels of tau influenced chemotherapy outcomes. In a study of breast cancer during stages I–III, tau was reported to be the most differentially expressed gene among the ones that are associated with preoperative paclitaxel-containing chemotherapy, and reduced tau mRNA increased the sensitivity of breast cancer cells to paclitaxel [173]. In a study on gastric cancer, the patients with tau-negative expression showed a significant response to paclitaxel administration [32]. These clinical studies indicated that the lower expression of tau positively influenced the chemotherapy outcomes, which may be used as a marker to select patients for paclitaxel therapy. However, a contradicting study reported that the expression of tau correlated much more closely with estrogen receptor (ER) expression instead of the sensitivity of paclitaxel, as tau contains an estrogen-response element upstream of its promoter [174], by assessing tau expression in primary breast cancer specimens of patients. They also conversely demonstrated that higher tau-positive is better associated with prognosis in patients treated with adjuvant paclitaxel chemotherapy and endocrine therapy. This inconsistent result may stem from interference with the ER status, as *MAPT* is a downstream target gene of the ER. In ER^+^ breast cancer, higher tau expression is an indicator of active estrogen signaling, rather to stabilize microtubule. Therefore, while high tau expression predicts insensitivity to paclitaxel (chemotherapy), it usually predicts a good response to endocrine therapy. This warrants further large-scale clinical study.

**Figure 7.**
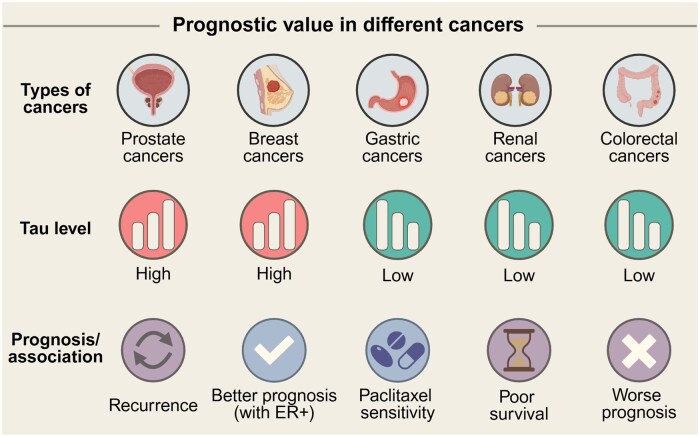
Tau in cancer biology. Tau expression exhibits distinct prognostic and therapeutic implications across cancers. In breast cancer, lower tau expression is associated with ER positivity and correlates with increased sensitivity to paclitaxel and a better prognosis. Prognostically, high tau levels are linked to recurrence in prostate cancer and a worse prognosis in colorectal cancer, while low tau expression correlates with paclitaxel sensitivity in gastric cancer and poor survival in renal cancer.

Tau may also have prognostic value in cancer. There were correlations between the Gleason score, an assessment for cancer differentiation, and tau expression in prostate cancer, providing evidence of tau as a prognostic recurrence indicator [33, 34]. In breast cancer, the level of tau can be used to identify patients who have limited resistance to chemotherapy, with ER-positive endocrine sensitivities. As to these patients, therefore, receiving adjuvant anthracyclines, paclitaxel chemotherapy, and endocrine therapy is associated with a better prognosis [174]. Oppositely, it has been reported that lower expression of *MAPT* has been correlated to poor overall survival after nephrectomy [35, 175], as well as the hypermethylation of the *MAPT* promoter CpG island was associated with a worse prognosis in patients with stage II colorectal cancer [35, 175]. Cumulatively, these observations demonstrate that tau expression is associated with cancer development and prognosis, while the influence depends on different types of cancer (Fig. 7). This is of great interest to tau biology studies since the mechanism of how tau participates in cancer has been insufficiently investigated.

## Discussions

It is unexpected to discover the functions of tau outside the brain, but even within the brain, several diseases, as discussed above, were not commonly associated with tau. Tau protein is expressed not only in the brain but also in peripheral organs, including the pancreas and heart [28, 29, 160, 161, 176, 177]. The evidence discussed here suggests that tau protein may play an important role in multiple organs throughout the body, which expands the understanding of the tau protein. However, it remains unclear whether the functions of tau in brain and peripheral diseases are alike. We conducted a comprehensive analysis of the functional differences of tau protein in the central nervous system and the periphery (Table 1). In the central nervous system, the molecular characteristic of tau protein pathology is the aggregation of specific isoforms. Physiologically, tau protein is mainly expressed in neurons; pathologically, tau protein expression is increased in glial cells (astrocytes and oligodendrocytes). In the CNS, tau function is highly concentrated on maintaining axonal transport, synaptic plasticity, and microtubule stability. In contrast, in the peripheral system, tau protein is functionally expressed in non-neuronal tissues (e.g., cardiac tissue and pancreatic islets) and various tumor tissues (e.g., breast cancer), participating in the regulation of insulin, iron, and beta-tubulin. In this context, peripheral tau protein dysfunction can independently drive pathology, for example, by contributing to metabolic disorders, insulin secretion, and chemotherapy resistance in malignant tumors, regardless of the pathological state of the brain. However, it is interesting to note that these regulatory functions observed are all related to the microtubule network, suggesting a conserved function of tau in the brain and the peripheral systems.

**Table 1.** Tau protein in the central nervous system and peripheral tissues.

	Central nervous system (CNS)	Peripheral tissues
**Major tau isoforms**	Pick’s disease (PiD): 3R-dominant Progressive supranuclear palsy (PSP): 4R-dominant Corticobasal degeneration (CBD): 4R-dominant Argyrophilic grain disease (AGD): 4R-dominant Globular glial tauopathy (GGT): 4R-dominant Aging-related Tau astrogliopathy (ARTAG): 4R-dominant Alzheimer’s disease (AD):3/4R	Often characterized by an imbalanced 3/4R ratio or functional overexpression in non-neuronal cells.
**Distribution**	Predominantly expressed in neurons; low or basal expression in microglia and astrocytes under homeostatic conditions.	Distributed in cardiac tissue, pancreatic islets, skeletal muscle, and malignant tissues (e.g., breast and prostate cancer).
**Physiological functions**	Axonal transport, microtubule stabilization, and synaptic plasticity	Microtubule stabilization, insulin secretion regulation, and cell motility
**Pathological drivers**	Driven primarily by Aβ deposition, neuroinflammation, oxidative stress, and *MAPT* mutations.	Driven by local microenvironmental factors such as hormonal fluctuations and metabolic stress.
**Biomarker implications**	Blood p-Tau levels reflect cerebral pathology.	Peripheral tau contributes to the systemic total tau pool.
**Peripheral tau influence**	CNS-derived spillover: a biomarker reflecting the “leakage” of proteins from damaged neurons into the circulation.	Independent driver: an active effector independent of CNS status.

Summarization of the differential characteristics of tau protein in the CNS versus peripheral tissues. Major isoforms, tissue distribution, physiological roles, pathological drivers, biomarker relevance, and mutual influences in different organs were discussed. The table highlights distinctions in tau expression between the two compartments and its roles in neuronal and systemic diseases.

T2DM is a common metabolic disorder and is closely associated with AD. In the brain, dysfunction of the tau protein has led to central insulin resistance and disturbances in glucose metabolism, providing a possible pathological mechanism for the link between cognitive impairment and diabetes [162, 163]. In pancreatic β-cells, abnormal tau expression directly interferes with insulin secretion and insulin sensitivity [27, 31], indicating that tau may additionally participate in the pathogenesis of diabetes. Thus, tau acts as a critical regulatory factor extensively involved in disrupting insulin metabolism in both the brain and pancreas. However, its mechanisms exhibit tissue specificity: in the brain, tau knockout impairs insulin signaling transduction, leading to neuronal insulin resistance and associated cognitive deficits, whereas in the pancreas, tau overexpression reduces insulin secretion, and tau knockout enhances insulin release and increases insulin sensitivity, with the mechanism of tau excessively stabilizing the microtubule network, physically hindering the transport and release of insulin vesicles. These findings collectively demonstrate tau as a central factor linking diabetes and neurological disorders, as well as a key bridge between the central nervous system and peripheral tissues. Therefore, further investigation into the differential roles of tau in central and peripheral tissues and elucidation of its underlying mechanisms are essential for understanding the comorbidity of diabetes and AD, and for developing novel tau-targeted therapies that simultaneously address diabetes and its neurological complications.

Interestingly, drugs targeting tau not only have great potential in AD treatment but also show promising therapeutic effects for other diseases. Salsalate, as a salicylate dimer, promotes tau degradation by reducing tau acetylation and is currently undergoing phase I clinical trials in AD patients. It also has a wide range of effects in other diseases and can be used as an effective anti-rheumatic molecule for arthritis treatment [178]. Additionally, it exhibits anti-inflammatory activity, lowers blood glucose levels, insulin resistance, and cytokine expression, making it suitable for research in the treatment of type 2 diabetes [31, 179]. Lonafarnib can alleviate tau pathology and behavioral impairment in FLTD mouse models and is also used in cancer treatment [180]. Meanwhile, GSK3 inhibitors effectively improve blood glucose control in diabetic mice, restore insulin secretion function, and reduce damage to pancreatic β-cells [181]. In the treatment of pancreatic ductal adenocarcinoma, GSK3β inhibitors (such as Elraglusib) in combination with chemotherapy drugs have shown significant efficacy, extending patient survival and improving objective response rates [182]. These findings indicate a possibility that tau acts multifunctionally in the body.

The broad involvement of tau protein across multiple physiological systems presents both significant opportunities and profound challenges for therapeutic development. While systemic tau reduction strategies, originally developed for tauopathies, may offer cross-disease benefits, such as mitigating ferroptotic damage in stroke or enhancing pancreatic insulin secretion in type 2 diabetes, they also carry substantial physiological risks. Evidence from tau-deficient models indicates that chronic tau depletion can lead to adverse outcomes, including cardiac hypertrophy, age-dependent hypertension, and even the paradoxical induction of central insulin resistance. The divergent roles, where tau reduction is beneficial in the pancreas but detrimental to cardiac and brain metabolic homeostasis, underscore the necessity for tissue-specific interventions over a pan-disease approach. Furthermore, realizing the translational potential of tau-targeted therapies requires overcoming formidable technical barriers. For central nervous system disorders, optimizing blood–brain barrier penetration for small molecules or biologics remains a primary hurdle, while in oncology, ensuring high tumor selectivity is essential to avoid exacerbating systemic toxicities or interfering with peripheral microtubule functions. The transition from preclinical models to clinical application is further complicated by the need for isoform-specific detection and targeting, ensuring that pathological tau species are eliminated while preserving the essential physiological functions of tau in peripheral organs.

For future studies, several critical avenues should be investigated. First, a fundamental priority is to delineate the “molecular switch” that governs the transition of tau from a physiological regulator to a pathological driver. Secondly, technological advancements are equally imperative to bridge current diagnostic gaps. The development of tissue-specific imaging modalities, such as novel PET tracers capable of monitoring tau dynamics in the heart or pancreas, and isoform-specific detection methods, will be indispensable for precision medicine. Furthermore, validating biomarkers like BD-tau across diverse clinical cohorts is a priority. Such validation will allow clinicians to distinguish central nervous system injury from peripheral tau fluctuations. Finally, future research should leverage cross-disease comparative studies to identify shared mechanistic nodes.

The intraneuronal aggregation of the protein tau in the form of tangles is widely described; however, in this review, we aimed to discuss the role of tau in diseases without tangle presentation. Notably, we focused on the functions of tau protein in cells other than neurons and in peripheral diseases. In nervous system diseases, including stroke, TBI, and MS, the genetic regulation, protein level, and phosphorylation state of tau are known to have a significant impact on disease progression. Tau is also associated with prognosis and drug sensitivity in cancer, is linked to the expression of insulin through phosphatase or kinase in diabetes, and is implicated in the angiotensin system in hypertension. Therefore, the therapeutic strategies targeting tau developed for AD and tauopathies may also be used for other diseases. We suggest that further research should focus on tau functions in specific cell types, organs, and diseases. In so doing, the fully appreciated landscape of tau functions might yield new therapeutic strategies for AD and other diseases.
